# Giant Preputial Calculus: The First Reported Case in Malaysia

**DOI:** 10.1155/2018/4606259

**Published:** 2018-09-18

**Authors:** Tze Huat Chong, Mohd Zuki Asyraf, Firdaus Hayati, Nornazirah Azizan, Nik Amin Sahid, Jesse Ron Swire Ting, Andee Dzulkarnaen Zakaria

**Affiliations:** ^1^Department of Surgery, Queen Elizabeth Hospital, Ministry of Health Malaysia, Kota Kinabalu, Sabah, Malaysia; ^2^Department of Surgery, Faculty of Medicine and Health Sciences, Universiti Malaysia Sabah, Kota Kinabalu, Sabah, Malaysia; ^3^Department of Pathobiology and Medical Diagnostics, Faculty of Medicine and Health Sciences, Universiti Malaysia Sabah, Kota Kinabalu, Sabah, Malaysia; ^4^Department of Urology, Queen Elizabeth Hospital, Ministry of Health Malaysia, Kota Kinabalu, Sabah, Malaysia; ^5^Department of Surgery, School of Medical Sciences, Universiti Sains Malaysia, Kelantan, Malaysia

## Abstract

Preputial calculus is a relative surgical rarity. It usually happens in elderly men with poor hygiene and uncircumcised penis complicated with phimosis. In the paediatric group, it is usually secondary to phimosis and other urologic and/or neurologic anomalies. Surgical treatment is the mainstay of treatment. Herein, we report a 27-year-old gentleman with preputial stone presented with obstructive uropathy and was successfully treated with surgical intervention. To the best of our knowledge, this is the first reported case of the largest preputial stone in Malaysia.

## 1. Introduction

Preputial calculus is a rare type of urolithiasis [1, 2]. Almost all the cases happen in elderly with poor hygiene and uncircumcised penis which are complicated with phimosis [1, 2]. However, it can also develop in children due to phimosis and other urologic and/or neurologic anomalies. Patients usually complain of obstructive uropathy, but less severe cases may manifest as a mass inside the prepuce, poor urine flow, strangury, hematuria, and foul smelling discharge.

The calculi are easily diagnosed via clinical examination as they are freely mobile within the preputial sac. They can exhibit as multiple as well as single stone as described in our reported case. Beside clinical assessment, a plain radiography is a useful modality to diagnose as well. The presence of radiopaque lesions proves their existences [1]. Once the diagnosis is confirmed, it is important to evaluate the rest of the urinary tract to unearth the presence of calculi from proximal urinary tract which may migrate to the narrowed passage such as the urethra and to some extent down to the phimotic prepuce [3].

Patient with preputial calculi should be treated surgically to prevent complications. Herein, we report a 27-year-old gentleman with preputial stone presented with obstructive uropathy and was successfully treated with surgical intervention. He underwent circumcision and removal of the calculus. To the best of our knowledge, this is the first reported case of largest preputial stone in a young adult in Malaysia.

## 2. Case Report

**Figure 1:**
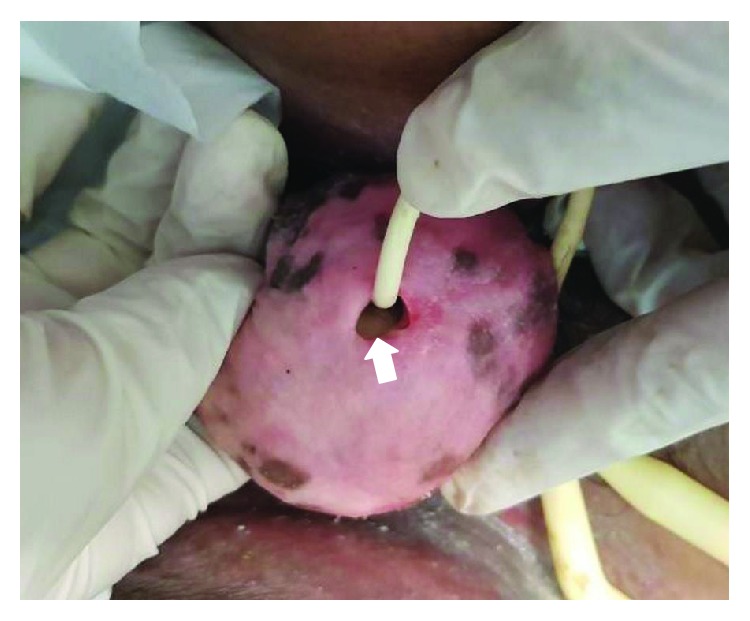
Tight phimosis with visible huge preputial stone (white arrow) seen within.

A 27-year-old male with underlying congenital hydrocephalus and paraplegia was admitted to the hospital due to infected sacral sore. He had a placement of a ventriculoperitoneal shunt when he was a child. Upon admission, he also complained of progressively having difficulty in passing urine and leaked urination but he denied dysuria, hematuria, and pyuria. On examination of his genitalia, the prepuce was deformed and enlarged with phimosis. To our surprise, there was a huge stony hard foreign body under the prepuce measuring 5 × 5 cm in size (Figure 1). It was visualised through the stenosed prepuce. Otherwise, the testes were normal and abdominal examination revealed no significant finding.

**Figure 2:**
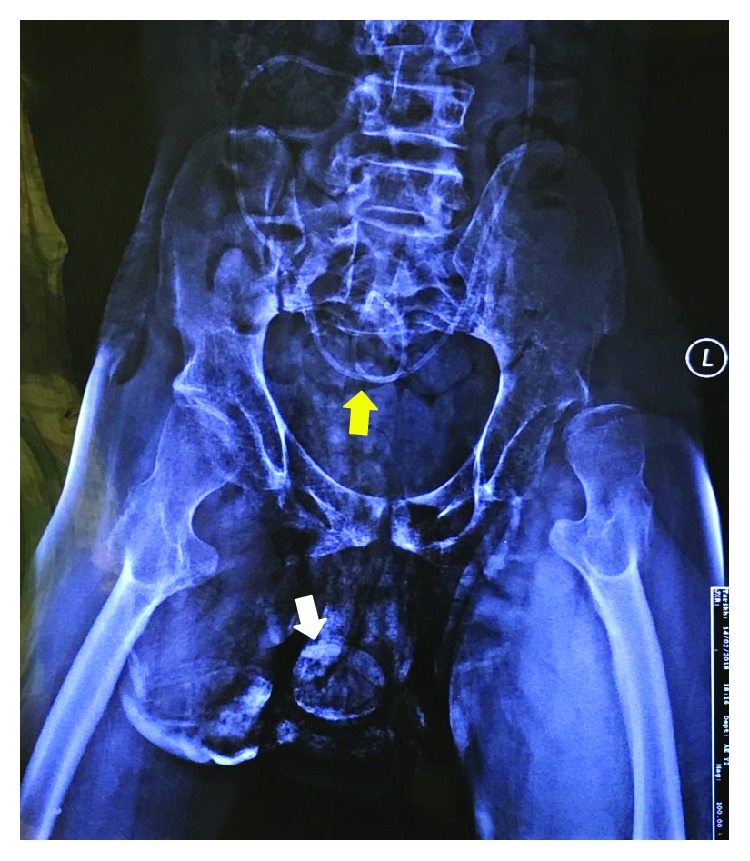
Pelvic radiograph showing the presence of well-rounded radiopaque calcification representing a preputial stone (white arrow), also the presence of VP shunt (yellow arrow) in the abdomen.

**Figure 3:**
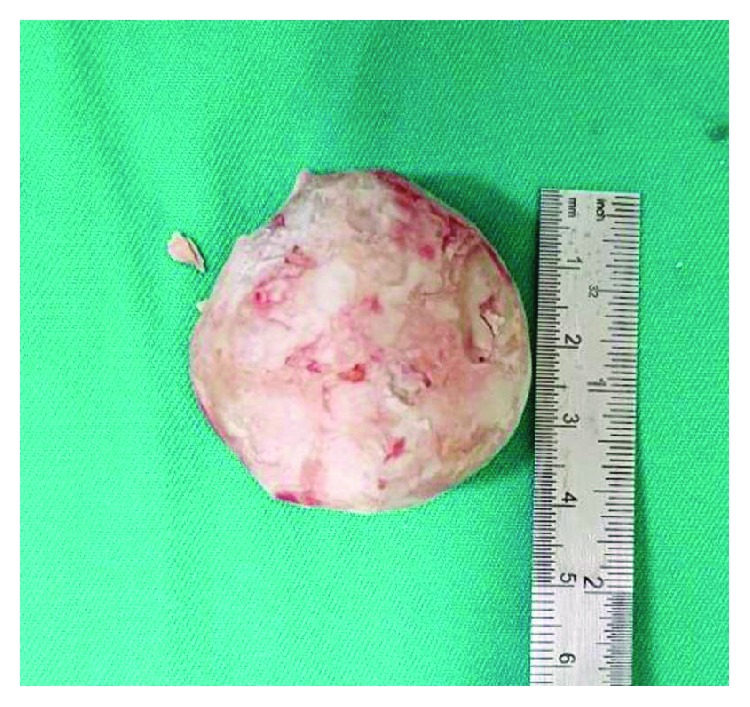
Closed up view of the preputial calculus measuring 4 × 4 cm after extraction.

**Figure 4:**
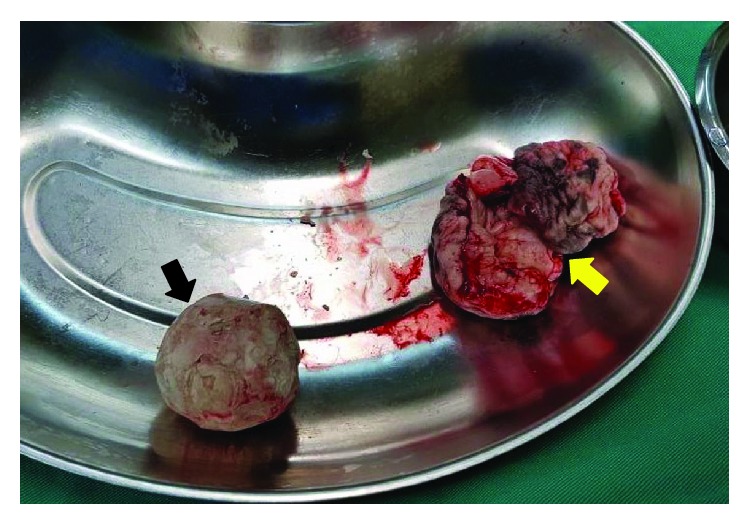
A single calculus (black arrow) and penile foreskin (yellow arrow) were retrieved after circumcision.

A pelvic radiograph was arranged and revealed a well-rounded radiopaque lesion in his penile region representing a large stone (Figure 2). Ultrasound of the kidney, ureter, and bladder (KUB) revealed an absence of proximal tract stone. A Foley's catheter was inserted to assist his urination, and it drained minimal amount of clear urine. His serum creatinine level was elevated initially but resolved after hydration. He was then planned for circumcision and removal of the preputial stone once his sacral sore improved. After almost a month in the ward, he finally underwent the circumcision. A flexible cystoscopic examination beforehand showed a trabeculated and small contracted bladder with no urethral stricture seen. The circumcision was undertaken using a dorsal slit technique. A huge preputial stone measuring 4 × 4 cm was uneventfully retrieved (Figures 3 and 4). His recovery went well without any complication postoperatively.

## 3. Discussion

Preputial stone is an unusual entity of the urologic field. It is hardly found during our clinical practice nowadays especially beyond 20^th^ century. Patients typically present with acute urinary retention or obstructive uropathy [1]. Only a few numbers of cases were accidentally found for other indication as in our reported case. There are scanty number of cases were described in the literatures after year 2000 as summarized in tabular form in Table 1.

It is postulated that the formation of preputial stone happens after (1) inspissated smegma in uncircumcised penis, (2) stasis with the precipitation of urinary salts, (3) struvite composition secondary to infection, or (4) trapped stones during migration from the proximal urinary tract calculi. In this reported case, the most likely cause is the combination of the first two theories. Uncircumcised penis has higher risk of smegma accumulation in the preputial sac especially among those who are rarely practising a clean hygiene. The smegma itself acts as a local irritant which can cause inflammation. Unsolved inflammatory process will lead to chronic inflammation and ultimately cause scarring of the prepuce.

This sac at the same time acts as a static reservoir for stone accumulation with metabolic action of urinary salt. It is common for stone to form in the prepuce especially among those with neurologic impairment and paraparesis or paraplegia as what has happened to our patient [4–6]. Moreover, urinary tract infection also precipitates stone formation particularly struvite type due to urease-producing bacteria [5]. This is evident by the growth of gram negative organisms such as *Escherichia coli*, *Enterococcus* sp., and *Citrobacter* sp. in urine culture of subpreputial space [1, 2].

Calculi are often palpable on examination of the prepuce; however, plain radiograph can confirm the existence. Ultrasound of KUB is essential to rule out any proximal stone as the treatment will include either shock wave, endoscopic or open surgery [3]. Any sonographic evidence of urinary obstruction warrants a bypass procedure such as endoscopic or percutaneous urinary stenting. By mere targeting the distal stone, it will not resolve the future dislodgement and reaccumulation of the urinary stone. Moreover, a metabolic evaluation should be ordered to investigate the causation of calculi formation especially in patient with calculi in any parts of the urinary tract. Unfortunately, we did not send our patient's stone for metabolic analysis since it is not routinely done in our center.

The mode of treatment involves removal of calculi and elimination of predisposing cause. As in this case, the patient underwent circumcision via dorsal slit procedure to remove the stone. Although preputial stone does not pose immediate life-threating event, it should be treated early and without delay [5]. Neglected preputial stone may cause serious morbidities such as hydronephrosis and renal failure secondary to obstructive uropathy and preputial skin fistula [6].

## 4. Conclusion

Knowledge on the importance of personal hygiene is utmost essential especially among uncircumcised individuals and neurological impairment. Encouragement on circumcision and emphasis on good hygiene should be disseminated to the public. Early detection and prompt action should be initiated early to prevent development of complications as mentioned above.

## Figures and Tables

**Table 1:** Comparison of our case with other literature reviews.

Authors/year of publication	Age (year)	Presenting complaint	Obstructive uropathy	Characteristics of calculi	Surgery
Present case	27	Sacral sore in chronic hydrocephalus and paraplegia	No	Single stone, 4 × 4 cm in size	Doral slit circumcision
Yuasa et al. (2001) [1]	92	Acute urinary retention with obstructive uropathy	Yes	Multiple sized stones weighing 100 gm	Doral slit circumcision
Bhat (2017) [2]	65	Lower urinary tract symptoms with obstructive uropathy	Yes	Multiple sized stones ranging from 0.4 to 1.5 cm	Doral slit circumcision
Spataru et al. (2015) [4]	5	Lower urinary tract symptoms with myelomeningocele	No	Single stone, 2 × 2 cm in size	Doral slit circumcision
Kekre et al. (2016) [5]	11	Lower urinary tract symptoms with meningomyelocele and placement of VP shunt	No	Multiple stones ranging from 0.3 to 2.5 cm	Doral slit circumcision
Tuğlu et al. (2013) [6]	12	Urinary tract infection with preputial skin fistula in a history of myelomeningocele operation	No	Multiple stones ranging from 1 to 2 cm in size	Doral slit circumcision
